# Milk metabolite profiling of dairy cows as influenced by mastitis

**DOI:** 10.3389/fvets.2024.1475397

**Published:** 2024-11-13

**Authors:** Chao Du, Xuehan Zhao, Shujun Zhang, Chu Chu, Xiaojian Zhang, Zhanwei Teng

**Affiliations:** ^1^College of Animal Science and Veterinary Medicine, Henan Institute of Science and Technology, Xinxiang, China; ^2^Key Lab of Agricultural Animal Genetics, Breeding and Reproduction of Ministry of Education, Huazhong Agricultural University, Wuhan, China

**Keywords:** mastitis, dairy cows, milk, metabolites, pathway analysis

## Abstract

Mastitis is a disease with frequent incidence in dairy cows, causing huge financial losses to the dairy industry globally. The identification of certain biomarkers is crucial for the early diagnosis and management of mastitis. Metabolomics technology is a useful tool to accurately and efficiently analyze the changes of metabolites in biofluids in response to internal and external stimulations. Milk is the secreted by udder, and milk metabolites can directly reflect whether the udder are in the healthy or diseased state. The milk metabolomics analysis of mastitis can reveal the physiological and pathological changes of mammary gland and screen the related biomarkers, so as to offer useful reference for the prediction, diagnosis, and management of mastitis. Therefore, the aim of the present study was to comprehensively summarize milk metabolic change caused by naturally occurring or experimentally induced mastitis in dairy cows. In addition, comparative analysis and enrichment analysis were conducted to further discover potential biomarkers of mastitis and to identify the relevant pathways differentiating the healthy and mastitic cows. Multiple milk metabolites were identified to be altered during mastitis based on different metabolomics platforms. It was noteworthy that there were 28 metabolites not only identified by at least two different studies, but also showed consistent change tendency among the different studies. By comparison with literature, we further identified 12 milk metabolites, including acetate, arginine, β-hydroxybutyrate, carnitine, citrate, isoleucine, lactate, leucine, phenylalanine, proline, riboflavin, and valine that were linked with the occurrence of mastitis, which suggested that these 12 milk metabolites could be potential biomarkers of mastitis in dairy cows. Several pathways were revealed to explain the mechanisms of the variation of milk metabolites caused by mastitis, such as phenylalanine, tyrosine and tryptophan biosynthesis, arginine and proline metabolism, riboflavin metabolism, and tricarboxylic acid (TCA) cycle. These results offer a further understanding for the alteration of milk metabolites caused by mastitis, which have a potential significance in the development of more reliable biomarkers for mastitic diagnosis in dairy cows.

## 1 Introduction

Mastitis, a mammary gland inflammation primarily caused by the co-infection of various pathogenic microorganisms, is a disease with frequent incidence in dairy cows. Generally, the incidence of bovine mastitis is 25%−60% globally (1, 2). Due to the decline in milk quality and production, mastitis has a negative economic effect on the dairy industry (3). The global economic losses from bovine mastitis is estimated at 35 billion dollars annually (4, 5). Even worse, mastitis is thought to be the main cause of overusing antibiotic, with many potential risks to consumers. Therefore, accurate diagnosis of mastitis and then effective treatment are crucial to the dairy industry.

The gold standard to diagnose bovine mastitis is bacteriological culturing, which aims to isolate the pathogens from infected milk samples (6). This method, however, has the disadvantage of being time-consuming. Because it entails plating milk samples on solid medium culture plates, incubating, assessing bacteria growth, and applying biochemical tests, which takes 2 to 7 days before results are available. Somatic cell count (SCC) can provide a clear numerical foundation for mastitic diagnosis. Multitudinous non-infectious factors such as parity, days in milk, and seasonal variations can also affect SCC, although these effects are always minimal (7). Therefore, additional effective diagnostic methods are desirable for bovine mastitis.

As an emerging omics technique, metabolomics is considered as the most comprehensive representation of an organism's physiological and biochemical status, and has been successfully used to investigate potential metabolite biomarkers in biofluids (8, 9). Milk is the secretion of udder, and changes of milk metabolic profiles directly reflects the mammary gland's healthy or diseased status in dairy cows (10). Furthermore, milk is available daily and milk collection is less harmful to dairy cows, and it is conductive to develop an in-line and non-invasive diagnosis technology by milk metabolomics detection for screening biomarkers. Therefore, milk metabolites are potential of interest as indicators for diagnosing mastitis in dairy cows, and many researchers put their eyes on the milk metabolic alterations by mastitis.

Currently, numerous differentially expressed milk metabolites have been identified between healthy and mastitic dairy cows. However, biomarkers that can be used for accurate diagnosis of mastitis are yet to be established. Moreover, little is known about the mechanisms by which mastitis alter milk metabolites. To address these issues, the primary objective of this study was to comprehensively review milk metabolites that were altered due to bovine mastitis. Furthermore, comparative analysis and then enrichment analysis were performed to discover potential biomarkers and to characterize relevant metabolic pathways of milk metabolic alterations caused by mastitis. The present study is crucial for further screening out reliable biomarkers that may facilitate the diagnosis of mastitis in dairy cows.

## 2 Differentially expressed milk metabolites between healthy and mastitic dairy cows

To date, numerous differentially expressed milk metabolites including amino acids, carbohydrates and derivates, nucleotides and analogs, nucleosides, and organic acid and derivates have been screened out between healthy and mastitic dairy cows. There are 12 previous studies that focus on identifying the differentially expressed milk metabolites according to the different definition of mastitis.

### 2.1 Differentially expressed milk metabolites identified by definition of mastitis by typical inflammation signs

Clinical mastitis is often characterized by a range of typical inflammation signs such as swelling and redness of udder, along with increased SCC. Using ultra-performance liquid chromatography-quadrupole-time of flight mass spectrometry, Xi et al. (11) detected 91 differential milk metabolites between healthy and mastitic cows classifying based on presentation of clinical signs and SCC. Using liquid chromatography-mass spectrometry (LC-MS), Wang et al. (12) uncovered 673 significantly different levels of milk metabolites between healthy and mastitic cows comprehensively judged according to the clinical manifestations of the udder, milk SCC, and the result of California mastitis test (CMT), known as a fast and convenient method to detect milk SCC. Using untargeted nuclear magnetic resonance spectroscopy (^1^H-NMR), Luangwilai et al. (13) found that acetate, alanine, benzoate, β-hydroxybutyrate, formate, hippurate, histidine, isoleucine, lactate, leucine, N-acetylamino acid, phenylalanine, threonine, valerate, and valine contents in milk significantly increased in mastitic cows selected by the result of CMT. Through ^1^H-NMR metabolomic approach, Zhu et al. (14) identified 22 milk molecules that were significantly different between healthy and mastitic cows judged based on the clinical manifestations of the udder and the result of CMT.

### 2.2 Differentially expressed milk metabolites identified by definition of mastitis by types of bacterial pathogens

Mastitis is commonly caused by the invasion of the mammary gland by bacterial pathogens. Hence, pathogens are often detected in mastitic cows' milk (15). Using gas chromatography-mass spectrometry, Hettinga et al. (16) reported that healthy cows' milk contained lower concentrations of volatile metabolites such as acetaldehyde, 2-butanone, butyric acid, 2-methylbutanal, and ethyl acetate compared with mastitic cows caused by coagulase negative staphylococci, *Escherichia coli, Staphylococcus aureus, Streptococcus dysgalactiae*, and *Streptococcus uberis*. Moyes et al. (17) showed that free glucose, lactose, and citrate were lower, whereas β-hydroxybutyrate and glucose-6-phosphate were higher when intramammary infection with *Escherichia coli* compared to pre-infection. Silanikove et al. (18) showed that milk lactic acid, glucose, and glucose-6-phosphate concentrations significantly changed due to mammary gland infected by several bacteria species, such as coagulase negative staphylococci, *Escherichia coli*, and streptococci. With the metabolomics approach of LC-MS, Thomas et al. (19) demonstrated that the change of milk metabolites by *Streptococcus uberis* infection were maximal at 81 h post challenge, and a total of 490 metabolites differentially expressed compared to the pre-challenge. Using gas chromatography time-of-flight mass spectrometry, Tong et al. (20) found 22 milk metabolites to be significantly different between the healthy and *Streptococcus agalactiae* mastitic cows. According to Bochniarz et al. (21), milk levels of kynurenine and tryptophan were significantly lower in mastitic cows due to the infection of algae from *Prototheca* group, compared to healthy animals.

### 2.3 Differentially expressed milk metabolites identified by definition of mastitis by SCC

With the increasing herd sizes of modern dairy industry, developing large-scale tools for identification of mastitis is of great interest to ensure adequate management. For this purpose, milk SCC have been globally adopted to manage udder health. By ^1^H-NMR, Sundekilde et al. (22) found that acetate, β-hydroxybutyrate, butyrate, isoleucine, and lactate significantly increased, while fumarate and hippurate significantly decreased in milk with high SCC (above 7.2 × 10^5^ cells/mL) compared to low SCC (below 1.4 × 10^4^ cells/mL). Most recently, differential somatic cell count (DSCC), which represents the proportion of neutrophils and lymphocytes over total SCC, has been introduced as a novel indicator of under health on a wide scale. Joint DSCC and SCC offer a more comprehensive depiction of under health status, enabling more precise differentiation between healthy and mastitic cows. By ^1^H-NMR based metabolomics, Bobbo et al. (23) revealed lower concentrations of carnitine, dimethylsulfone, galactose, galactose-1-phosphate, glucose, hippurate, lactose, lecithin, orotate, riboflavin, and succinate, and higher levels of acetate, choline, lactate, phenylalanine, O-acetylcarnitine, valine, and 2-oxoglutarate in milk with high SCC and DSCC (SCC ≥ 200,000 cells/mL and DSCC ≥ 60%) than low SCC and DSCC (SCC <200,000 cells/mL and DSCC <60%).

## 3 The same milk metabolites identified by different references

Although a lot of differential milk metabolites have been identified, in the present study, the same metabolites identified by different studies are paid much attention to. That is, if a metabolite is identified by one article, when it is also identified by the other articles, then it will be used for further analysis. By comparison analysis, there were 41 milk metabolites that were identified by at least 2 different references. For example, valine was identified by Luangwilai et al. (13), Zhu et al. (14), and Bobbo et al. (23), and acetate was identified by Luangwilai et al. (13), Sundekilde et al. (22), and Bobbo et al. (23).

### 3.1 The milk metabolites of inconsistent change tendency and possible reasons

Some metabolites showed inconsistent change tendency among the various references. For example, Thomas et al. (19) and Tong et al. (20) reported that the concentration of uridine in milk significantly increase in mastitic cows. Conversely, the significant decrease of milk uridine from mastitic cows was observed in the studies of Xi et al. (11) and Wang et al. (12). There are many reasons to account for this phenomenon. One of the possible reasons is the particular time point of infection over the course of mastitis. For example, compared to pre-challenge, the content of guanine in milk showed significant increase at 57 h post challenge with *Streptococcus uberis*, however, its concentration significantly decreased at 312 h post challenge (19). Different metabolomics platforms might be another reason to explain the phenomenon that the same milk metabolites showed inconsistent change tendency among different references. For example, in the study of Luangwilai et al. (13), ^1^H-NMR based metabolomics investigation was applied, whereas the metabolomics technique of LC-MS was used in the study of Wang et al. (12), which might lead to the opposite tendency of threonine between these 2 studies. Zhu et al. (14) judged mastitic cows based on the combination of clinical manifestations of the udder and CMT, whereas Luangwilai et al. (13) determined the presence of mastitis only based on CMT, which is a semiquantitative measure, and its interpretation may be subjective, leading to results that are not so accurate (24). That way, the different definitions of mastitis may contribute to the opposite change tendency of *N*-acetylglucosamine in these 2 studies. Besides, the differences in dairy cow breeds, number of parity, days in milk, and the varieties in bacterial types causing mastitis may also contribute to the discrepancy in results. For instance, Chinese Holstein cows with 2–4 parity and 150–195 days in milk were used in the study of Zhu et al. (14), while in the study of Bobbo et al. (23), Simmental cows with average 3.8 parities and 219 days in milk were used, which may result in the inconsistent change tendency of 2-oxoglutarate. Dairy cows with *Streptococcus uberis* mastitis (19) and mastitic dairy cows infected with *Streptococcus agalactiae* (20) could be one of the possible reasons for the opposite result of asparagine. Mastitis was naturally occurrent in the study of Wang et al. (12), whereas mastitis was experimentally induced in the study of Thomas et al. (19), which may cause the inconsistent change tendency of nonadecanoic acid in these 2 studies. Table 1 lists the information of tendency and fold change of these milk metabolites between healthy and mastitic cows, and also comprehensively summarize the possible factors contributing to this phenomenon.

**Table 1:** The milk metabolites that showed inconsistent change tendency among different references and the possible reasons.

**Metabolites**	**References**	**Tendency**	**Fold change**	**The possible factors for inconsistent change tendency**					
				**Metabolomics platforms**	**Methods for determining mastitis**	**Sampling time points during inflammation**	**Bacterial types**	**Cows**	**Types of mastitis**
Asparagine	Thomas et al. (19)	↑	56.08	LC-MS	Bacterial pathogens	0, 36, 42, 57, 81, and 312 h post-challenge	*Streptococcus uberis*	Holstein cows, 1 to 5 parity, 60 to 160 days in milk	Experimentally induced
	Tong et al. (20)	↓	0.50	GC-TOF MS	Bacterial pathogens	/	*Streptococcus agalactiae*	2.63 ± 0.38 parity, 154.60 ± 7.58 days in milk	Naturally occurrent
Choline	Thomas et al. (19)	↓	0.10	LC-MS	Bacterial pathogens	0, 36, 42, 57, 81, and 312 h post-challenge	*Streptococcus uberis*	Holstein cows, 1 to 5 parity, 60 to 160 days in milk	Experimentally induced
	Bobbo et al. (23)	↑	1.41–2.00	^1^H-NMR	SCC, DSCC	/	/	Simmental cows, 2 to 6 parity, 103 to 356 days in milk	/
Glucose	Xi et al. (11)	↓	0.24	UPLC-Q-TOF MS	Inflammation signs, SCC	/	/	Holstein cows, 2 to 4 parity, 3 to 7 month of lactation stage	Naturally occurrent
	Moyes et al. (17)	↑	~1.27	Fluorometric method	Bacterial pathogens	−180, −132, −84, −36, −12, 12, 24, 36, 48, 60, 72, 84, 96, 132, and 180 h relative to challenge	*Escherichia coli*	Primiparous Holstein cow, ~4 to 6 weeks in lactation	Experimentally induced
	Silanikove et al. (18)	↓	~0.43	Enzymatic reactions	Bacterial pathogens	/	coagulase negative staphylococci, *Escherichia coli*, and streptococci	Holstein cow	Experimentally induced
	Thomas et al. (19)	↓	0.10	LC-MS	Bacterial pathogens	0, 36, 42, 57, 81, and 312 h post-challenge	*Streptococcus uberis*	Holstein cows, 1 to 5 parity, 60 to 160 days in milk	Experimentally induced
	Bobbo et al. (23)	↓	0.71–1.00	^1^H-NMR	SCC, DSCC	/	/	Simmental cows, 2 to 6 parity, 103 to 356 days in milk	/
Glucose-6-phosphate	Moyes et al. (17)	↑	~3.00	Enzymatic-fluorometric method	Bacterial pathogens	−180, −132, −84, −36, −12, 12, 24, 36, 48, 60, 72, 84, 96, 132, and 180 h relative to challenge	*Escherichia coli*	Primiparous Holstein cow, ~4 to 6 weeks in lactation	experimentally induced
	Silanikove et al. (18)	↓	~0.67	Enzymatic reactions	Bacterial pathogens	/	coagulase negative staphylococci, *Escherichia coli*, and streptococci	Holstein cow	Experimentally induced
	Tong et al. (20)	↓	0.34	GC-TOF MS	Bacterial pathogens	/	*Streptococcus agalactiae*	2.63 ± 0.38 parity, 154.60 ± 7.58 days in milk	Naturally occurrent
Guanine	Thomas et al. (19)	↑	9.59	LC-MS	Bacterial pathogens	0, 36, 42, 57, 81, and 312 h post-challenge	*Streptococcus uberis*	Holstein cows, 1 to 5 parity, 60 to 160 days in milk	Experimentally induced
	Tong et al. (20)	↓	0.33	GC-TOF MS	Bacterial pathogens	/	*Streptococcus agalactiae*	2.63 ± 0.38 parity, 154.60 ± 7.58 days in milk	Naturally occurrent
Hippurate	Xi et al. (11)	↓	0.26	UPLC-Q-TOF MS	Inflammation signs, SCC	/	/	Holstein cows, 2 to 4 parity, 3 to 7 month of lactation stage	Naturally occurrent
	Luangwilai et al. (13)	↑	2.34	^1^H-NMR	CMT	/	/	Crrssbred Hostein cows, 2 to 4 lactation cycle, mid-lactation period	Naturally occurrent
	Thomas et al. (19)	↓	0.55	LC-MS	Bacterial pathogens	0, 36, 42, 57, 81, and 312 h post-challenge	*Streptococcus uberis*	Holstein cows, 1 to 5 parity, 60 to 160 days in milk	Experimentally induced
	Sundekilde et al. (22)	↓	~0.80	^1^H-NMR	SCC	/	/	Danish HolsteinFriesian and Jersey cows	/
	Bobbo et al. (23)	↓	0.71-1.00	^1^H-NMR	SCC, DSCC	/	/	Simmental cows, 2 to 6 parity, 103 to 356 days in milk	/
*N*-acetylglucosamine	Luangwilai et al. (13)	↑	2.19	^1^H-NMR	CMT	/	/	Crrssbred Hostein cows, 2 to 4 lactation cycle, mid-lactation period	Naturally occurrent
	Zhu et al. (14)	↓	0.54	^1^H-NMR	Inflammation signs, CMT	/	/	Holstein cows, 2 to 4 parity, 150 to 195 days in milk	Naturally occurrent
Nonadecanoic acid	Wang et al. (12)	↓	0.37	LC-MS	Inflammation signs, SCC, CMT	/	/	2 to 4 parity, 156 to 191 days in milk	Naturally occurrent
	Thomas et al. (19)	↑	12.01	LC-MS	Bacterial pathogens	0, 36, 42, 57, 81, and 312 h post-challenge	*Streptococcus uberis*	Holstein cows, 1 to 5 parity, 60 to 160 days in milk	Experimentally induced
O-Acetylcarnitine	Zhu et al. (14)	↓	0.27	^1^H-NMR	Inflammation signs, CMT	/	/	Holstein cows, 2 to 4 parity, 150 to 195 days in milk	Naturally occurrent
	Thomas et al. (19)	↓	0.07	LC-MS	Bacterial pathogens	0, 36, 42, 57, 81, and 312 h post-challenge	*Streptococcus uberis*	Holstein cows, 1 to 5 parity, 60 to 160 days in milk	Experimentally induced
	Bobbo et al. (23)	↑	1.00-1.41	^1^H-NMR	SCC, DSCC	/	/	Simmental cows, 2 to 6 parity, 103 to 356 days in milk	/
Threonine	Wang et al. (12)	↓	0.35	LC-MS	Inflammation signs, SCC, CMT	/	/	2 to 4 parity, 156 to 191 days in milk	Naturally occurrent
	Luangwilai et al. (13)	↑	2.88	^1^H-NMR	CMT	/	/	Crrssbred Hostein cows, 2 to 4 lactation cycle, mid-lactation period	Naturally occurrent
Uridine	Xi et al. (11)	↓	0.27	UPLC-Q-TOF MS	Inflammation signs, SCC	/	/	Holstein cows, 2 to 4 parity, 3 to 7 month of lactation stage	Naturally occurrent
	Wang et al. (12)	↓	0.47	LC-MS	Inflammation signs, SCC, CMT	/	/	2 to 4 parity, 156 to 191 days in milk	Naturally occurrent
	Thomas et al. (19)	↑	1.77	LC-MS	Bacterial pathogens	0, 36, 42, 57, 81, and 312 h post-challenge	*Streptococcus uberis*	Holstein cows, 1 to 5 parity, 60 to 160 days in milk	Experimentally induced
	Tong et al. (20)	↑	4.57	GC-TOF MS	Bacterial pathogens	/	*Streptococcus agalactiae*	2.63 ± 0.38 parity, 154.60 ± 7.58 days in milk	Naturally occurrent
2-oxoglutarate	Zhu et al. (14)	↓	0.14	^1^H-NMR	Inflammation signs, CMT	/	/	Holstein cows, 2 to 4 parity, 150 to 195 days in milk	Naturally occurrent
	Bobbo et al. (23)	↑	1.00-1.41	^1^H-NMR	SCC, DSCC	/	/	Simmental cows, 2 to 6 parity, 103 to 356 days in milk	/
5-Hydroxy-l-tryptophan	Xi et al. (11)	↓	0.01	UPLC-Q-TOF MS	Inflammation signs, SCC	/	/	Holstein cows, 2 to 4 parity, 3 to 7 month of lactation stage	Naturally occurrent
	Thomas et al. (19)	↑	30.27	LC-MS	Bacterial pathogens	0, 36, 42, 57, 81, and 312 h post-challenge	*Streptococcus uberis*	Holstein cows, 1 to 5 parity, 60 to 160 days in milk	Experimentally induced

Upward arrows (↑) indicate that the metabolites are higher in mastitic cows' milk compared to milk from healthy cows; downward arrows (↓) mean that the metabolites are lower in mastitic cows' milk compared to milk from healthy cows.

“/” means that the relative contents are not applicable or not been demonstrated in the references.

### 3.2 The milk metabolites of consistent change tendency

Of the aforementioned 41 milk metabolites, there were 28 metabolites that showed consistent change tendency among the various references, including acetate, adenine, arginine, β-hydroxybutyrate, capryloylcholine, carnitine, *cis*-aconitate, citrate, creatine, creatinine, DL-2-aminooctanoic acid, girgensonine, glycerol, indoleacrylic acid, isoleucine, lactate, leucine, orotate, phenylalanine, piperidine, proline, puromycin, riboflavin, sn-glycero-3-phosphocholine, sn-glycero-3-phosphoethanolamine, valine, 6-Hydroxy-5-methoxyindole glucuronide, and 8-Hydroxy-7-methylguanine. Table 2 lists the information of tendency and fold change of these milk metabolites between healthy and mastitic cows.

**Table 2:** The milk metabolites that showed consistent change tendency among different references.

**Metabolites**	**References**	**Tendency**	**Fold change**
Acetate	Luangwilai et al. (13)	↑	2.57
	Sundekilde et al. (22)	↑	~5.50
	Bobbo et al. (23)	↑	1.00–1.41
Adenine	Wang et al. (12)	↓	0.45
	Thomas et al. (19)	↓	0.32
Arginine	Xi et al. (11)	↑	6.72
	Zhu et al. (14)	↑	8.87
	Thomas et al. (19)	↑	4.44
β-hydroxybutyrate	Moyes et al. (17)	↑	~2.25
	Sundekilde et al. (22)	↑	~1.80
Capryloylcholine	Xi et al. (11)	↓	Infinitesimal
	Wang et al. (12)	↓	0.48
Carnitine	Xi et al. (11)	↓	0.36
	Zhu et al. (14)	↓	0.06
	Thomas et al. (19)	↓	0.16
	Bobbo et al. (23)	↓	0.71–1.00
*cis*-Aconitate	Xi et al. (11)	↓	0.46
	Zhu et al. (14)	↓	0.23
	Thomas et al. (19)	↓	0.04
Citrate	Xi et al. (11)	↓	0.35
	Zhu et al. (14)	↓	0.18
	Moyes et al. (17)	↓	~0.73
Creatine	Zhu et al. (14)	↓	0.25
	Thomas et al. (19)	↓	0.20
Creatinine	Zhu et al. (14)	↓	0.21
	Thomas et al. (19)	↓	0.29
DL-2-aminooctanoic acid	Wang et al. (12)	↓	0.36
	Thomas et al. (19)	↓	0.07
Girgensonine	Wang et al. (12)	↑	15.87
	Thomas et al. (19)	↑	499.30
Glycerol	Thomas et al. (19)	↑	4.52
	Tong et al. (20)	↑	5.14
Indoleacrylic acid	Wang et al. (12)	↑	15.87
	Thomas et al. (19)	↑	14.47
Isoleucine	Xi et al. (11)	↑	9.21
	Wang et al. (12)	↑	infinite
	Luangwilai et al. (13)	↑	3.09
	Zhu et al. (14)	↑	18.61
	Sundekilde et al. (22)	↑	~1.50
Lactate	Luangwilai et al. (13)	↑	2.57
	Zhu et al. (14)	↑	39.54
	Thomas et al. (19)	↑	75.26
	Sundekilde et al. (22)	↑	~12.00
	Bobbo et al. (23)	↑	2.00–2.83
Leucine	Luangwilai et al. (13)	↑	2.40
	Zhu et al. (14)	↑	4.79
	Thomas et al. (19)	↑	8.67
Orotate	Thomas et al. (19)	↓	0.03
	Bobbo et al. (23)	↓	0.71–1.00
Phenylalanine	Luangwilai et al. (13)	↑	2.82
	Thomas et al. (19)	↑	30.10
	Bobbo et al. (23)	↑	1.41–2.00
Piperidine	Wang et al. (12)	↑	2.83
	Thomas et al. (19)	↑	5.02
Proline	Xi et al. (11)	↑	3.11
	Zhu et al. (14)	↑	33.13
	Thomas et al. (19)	↑	5.15
Puromycin	Wang et al. (12)	↑	6.80
	Thomas et al. (19)	↑	3,061.39
Riboflavin	Thomas et al. (19)	↓	0.31
	Bobbo et al. (23)	↓	0.18–0.25
sn-glycero-3-phosphocholine	Xi et al. (11)	↓	0.43
	Wang et al. (12)	↓	0.14
	Thomas et al. (19)	↓	0.03
sn-glycero-3-phosphoethanolamine	Wang et al. (12)	↓	0.39
	Thomas et al. (19)	↓	0.02
Valine	Xi et al. (11)	↑	10.24
	Luangwilai et al. (13)	↑	2.63
	Zhu et al. (14)	↑	8.25
	Bobbo et al. (23)	↑	1.00–1.41
6-Hydroxy-5-methoxyindole glucuronide	Wang et al. (12)	↑	2.71
	Thomas et al. (19)	↑	102.46
8-Hydroxy-7-methylguanine	Wang et al. (12)	↓	0.40
	Thomas et al. (19)	↓	0.07

Upward arrows (↑) indicate that the metabolites are higher in mastitic cows' milk compared to milk from healthy cows; downward arrows (↓) mean that the metabolites are lower in mastitic cows' milk compared to milk from healthy cows.

All these milk metabolites were only identified by the method of metabolomics. To further illuminate the relationships between these metabolites and mastitis, literature search was conducted, and 12 milk metabolites, including acetate, arginine, β-hydroxybutyrate, carnitine, citrate, isoleucine, lactate, leucine, phenylalanine, proline, riboflavin, and valine were confirmed to be closely associated with immune function and mammary infection.

The correlation between *Staphylococcus aureus* load and the contents of milk isoleucine and leucine was strong. In addition, a high sensitivity and specificity of these two amino acids, at a threshold of 100 μg/ml, to discriminate *Staphylococcus aureus* positive and *Staphylococcus aureus* negative milk samples was observed (25). *Staphylococcus aureus* requires isoleucine, leucine, and valine to synthesize protein for nutrients requirements, which is vital to *Staphylococcus aureus*'s development and virulence (26). Isoleucine levels affected the expression of genes linked to the capacity of *Staphylococcus aureus* to cause disease (27). Accoridng to Konashi et al. (28), phenylalanine deficiency in chickens impaired their immunological responses, while dietary supplementation with this amino acid could reverse the problem. In addition, valine, phenylalanine, isoleucine, leucine, and proline in the serum showed high predictive abilities for discriminating the mastitis from healthy cows (1, 29). Carnitine transports long-chain fatty acids from the cytosol to the mitochondrial matrix, which in turn, further regulates the energy metabolism. Early lactation excessive energy deficit impairs immune function, which has been linked to a higher risk of infectious conditions such as mastitis (30). Carnitine and proline in serum pre-calving could correctly classified cows for their future mastitic status (31). Arginine is essential for the maintenance of immune function, and this amino acid exerts anti-inflammatory functions. In primary bovine mammary epithelial cells, arginine supply reduced the proinflammatory responses induced by lipopolysaccharide (LPS) via regulating the amount of proinflammatory cytokines and chemokines as well as the activity of NF-κB (32).

Riboflavin, also known as vitamin B_2_, has a variety of immune modulatory effects. It has been demonstrated that intramuscular administration of riboflavin in cows stimulates neutrophil function such as phagocytic bactericidal activity and nitroblue tetrazolium reductivity. Besides, riboflavin injection intravenously to cows with high SCC caused a rapid reduction of SCC in udder quarters infected by *Staphylococcus aureus* (33, 34). Previous studies showed a correlation between blood β-hydroxybutyrate and the likelihood of mastitis in early lactation (35). Increased β-hydroxybutyrate levels are linked to oxidative stress, which impairs the immune system and intensifies the inflammation. Besides, increased β-hydroxybutyrate levels inhibit bovine peripheral blood mononuclear cells, and impair polymorphonuclear leukocytes activity, exposing dairy cows to mastitis in the periparturient phase (36). Recent investigations reveal that β-hydroxybutyrate is metabolized in reaction to immune challenges. According to Gross et al. (37), the level of β-hydroxybutyrate in cows' blood was linked to their metabolic responses to an LPS challenge and to their subsequent recovery of udder health and function. The major end products of carbohydrate metabolism are acetate and lactate, which are created by milk bacteria, or by anaerobic epithelium respiration in an oxygen-deprived environment following mastitis. The presence of bacteria in milk will produce a distinct metabolic fingerprint, expressed as an increase in lactate concentration (22, 23). Citrate is regarded as a biomarker of mitochondrial metabolism in the mammary gland. Citrate is secreted into milk by the mammary epithelial cells, and the concentration of this substance indicates mammary activity (17, 38).

All these evidences confirm the changes of acetate, arginine, β-hydroxybutyrate, carnitine, citrate, isoleucine, lactate, leucine, phenylalanine, proline, riboflavin, and valine are linked to mammary infection, suggesting these 12 milk metabolites may be potential biomarkers for diagnosing mastitis in cows.

## 4 Metabolic pathways analysis of milk metabolites affected by mastitis

To gain a deeper understanding of how multiple pathways distinguished the healthy and mastitic milk, the 28 metabolites which showed consistent change tendency were used as a basis for a Kyoto Encyclopedia of Genes and Genomes (KEGG) enrichment analysis by using MetaboAnalyst 5.0. Pathway topology analysis with a criterion of *p* < 0.05 and impactor value >0.1 revealed 4 indicated pathways, namely phenylalanine, tyrosine and tryptophan biosynthesis, arginine and proline metabolism, riboflavin metabolism, and tricarboxylic (TCA) cycle (Table 3).

**Table 3:** Metabolic pathways revealed by enrichment analysis on the milk metabolites significantly differing the healthy dairy cows from the mastitic ones (*p* value <0.05 and impact value >0.1).

**Metabolic pathways**	**Metabolites**	***p* value**	**Impactor value**
Arginine and proline metabolism	Arginine, Creatine, Proline	0.0065	0.15
Citrate cycle (TCA cycle)	cis-Aconitate, Citrate	0.018	0.14
Phenylalanine, tyrosine and tryptophan biosynthesis	Phenylalanine	0.042	0.50
Riboflavin metabolism	Riboflavin	0.042	0.50

Phenylalanine is needed to maintain an enough provision of tetrahydrobiopterin to produce nitric oxide (NO) by inducible nitric oxide synthase in activated macrophages and other leukocytes, whereas NO is a pro-inflammatory cytokine with important regulatory functions in the inflammatory process when bacteria invade the mammary gland (39). Arginine is the main amino acid to synthesize creatine in the liver. Since creatine is an intermediary molecule in energy processes, it has significant importance on regulating dairy cows' negative energy balance (40, 41). During the periparturient stage, NEB is a major contributor to oxidative stress, which may impair immune and anti-inflammatory responses. According to a recent study, oxidative stress is the main cause of periparturient diseases in dairy cows, including mastitis (42). Proline is a crucial amino acid that protects lymphocytes from apoptosis, stimulates cell growth, and promotes antibody generation (43). The increase of free amino acids including arginine, proline, and phenylalanine in mastitic milk may be linked to increase of pathogen-specific fermentative processes and protein degradation activities.

It has been discovered that riboflavin increases the non-specific host defense mechanisms in mice against a range of bacterial infections by inducing neutrophil production and improving macrophage activity. In mastitic cows, the riboflavin metabolism may be linked to the improvement of peripheral blood neutrophils' nitroblue tetrazolium reductivity and the activation of host-defense mechanisms against bacterial infection in the mammary gland (33, 34). Neutrophils are the main cells of defense involved in bacterial clearance after mammary gland infection. During the peripartum stage, impairment of neutrophil activity is a key contributor to the high incidence of mastitis, and postpartum mastitis also have been associated with decreased neutrophil function (44). The TCA cycle, which is the fundamental metabolic pathway in mitochondria, is vital for dairy cows' health. By disturbing the TCA cycle in the mammary gland, mastitis alters milk metabolites, and the pathogens are expected to divert these for their own metabolic processes and development as the mastitis advances. The levels of some TCA cycle intermediates such as *cis*-aconitate and citrate decreased significantly in the mastitic milk, suggesting that the TCA cycle is downregulated in bovine mastitis. Besides, citrate generates a citrate-iron complex that is accessible to bacteria by competing with lactoferrin for iron. The notable decrease of citrate in mastitic cows may suggest that lactoferrin's ability to bind iron has increased, contributing to host defense and limiting availability of iron to bacterial invaders (17, 38).

As a result, our results offer possible molecular mechanisms for the observed variations in milk metabolomes between the healthy and mastitic dairy cows.

## 5 Conclusions

**Figure 1:**
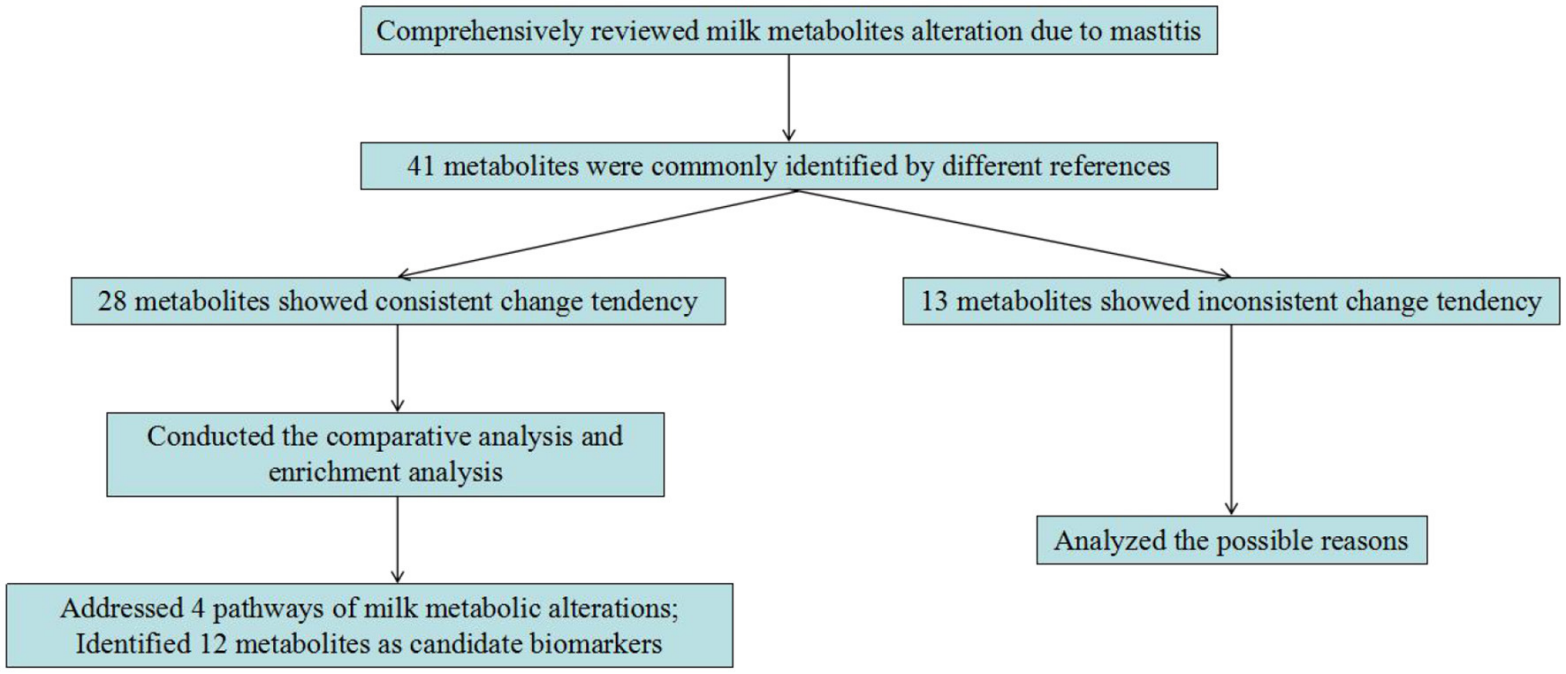
The main content of this study.

In dairy cows, mastitis is a complicated and serious syndrome causing large financial losses in the dairy industry. Thus, the development of diagnostic biomarkers for mastitis is highly desired. This study conducted a comprehensive overview of the application of metabolomics technology on screening the milk metabolic change in response to mastitis. Numerous metabolites in milk were altered due to mastitis. By comparative analysis, we found 28 metabolites with consistent change tendency among the different references. These metabolites were mainly involved in phenylalanine, tyrosine and tryptophan biosynthesis, arginine and proline metabolism, riboflavin metabolism, and TCA cycle. By further comparison with literature, several metabolites including acetate, arginine, β-hydroxybutyrate, carnitine, citrate, isoleucine, lactate, leucine, phenylalanine, proline, riboflavin, and valine were confirmed to be involved in inflammation. Therefore, the present study highlights that these 12 milk metabolites may be used as biomarkers to diagnose mastitis in dairy cows (Figure 1). It merits further experiments to investigate how useful of these metabolites as biomarkers for mastitis diagnosis, and also its biochemical and physiological mechanisms in the occurrence of mastitis.

As is well documented that various bacterial species that cause mastitis may influence a distinct metabolomic profile. However, the present study only generally summarized the milk metabolites alteration response to mastitis, and did not classify according to the types of bacterial pathogens. In the future, the study of milk metabolomics should focus more on the basis of etiologic agent, which will help for identifying biomarkers of mastitis against the specific bacterial types.
